# Bilateral testicular torsion in an adolescent: a case with challenging diagnosis

**DOI:** 10.1590/S1677-5538.IBJU.2017.0371

**Published:** 2018

**Authors:** L. Lorenzo, E. Martínez-Cuenca, E. Broseta

**Affiliations:** 1 Hospital Universitario y Politécnico La Fe, Valencia, Espanha

**Keywords:** Testis, Spermatic Cord Torsion, Adolescent

## Abstract

Bilateral testicular torsion is a very uncommon emergency, with a challenging differential diagnosis. We describe the case of a 15-year-old patient with a left testicular torsion of 48 hours of duration and a sudden onset of right scrotum pain during his stay at the emergency area. Bilateral testicular torsion was diagnosed after repeat physical examination and doppler ultrasound, which had been normal for right testis in a first evaluation. Surgical exploration was performed with orchiectomy in left testis and fixation in right testis. In previous literature, there are reported bilateral torsion only in four adolescents and five adults. With this case, we demonstrate that bilateral spermatic cord torsion may be easily overlooked in a patient with acute scrotum and we emphasize the importance of bilateral exploration in testicular torsion.

## INTRODUCTION

The incidence of testicular torsion in patients under 25 years of age is estimated at around 1 per 4000 (1). However, bilateral testicular torsion is an extremely rare entity. In this report, we present a particular case of early metachronous bilateral testicular torsion in an adolescent and we also carry out a literature review of this particular condition in adolescents and adults.

## CASE REPORT

**Figure 1:**
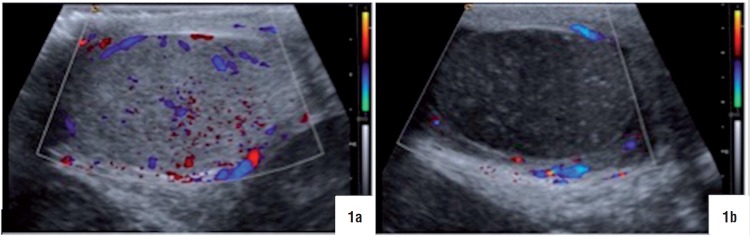
Doppler ultrasound in the initial evaluation. a) right testis. b) left testis.

A 15-year-old patient was admitted to our emergency department (E.D.) with a left hemi-scrotal pain, tenderness and swelling of 48 hours of duration. The pain was associated to nausea, one episode of vomiting and fever (37.4°C). There was no previous history of trauma or lower urinary tract symptoms. Moreover, the patient denied any similar episodes or right scrotal pain at that moment. On physical examination, left scrotum was very swollen, highly erythematous and tender on palpation, which made the assessment of its contents very difficult. Right testis evaluation was normal. White blood count was 16.300 per microliter and urinalysis was normal. Color doppler ultrasound revealed a twisted left cord, absent vascular flow, heterogeneous testicular structure and reactive hydrocele in left scrotum. Right testicular sonography was completely normal, with presence of a standard vascular flow (Figure-1).

With the diagnosis of non-salvageable left testicular torsion, we decided to carry out a delayed orchiectomy. However, two hours later, while the patient was in the observation ward receiving analgesic treatment, he presented a sudden right testis pain. At physical re-evaluation, right testicle was lying transversely and retracted, without inflammatory signs but painful on palpation. Therefore, we repeated the ultrasound study, finding absence of vascular flow at the right testicle, with no additional changes in the left testicle.

**Figure 2:**
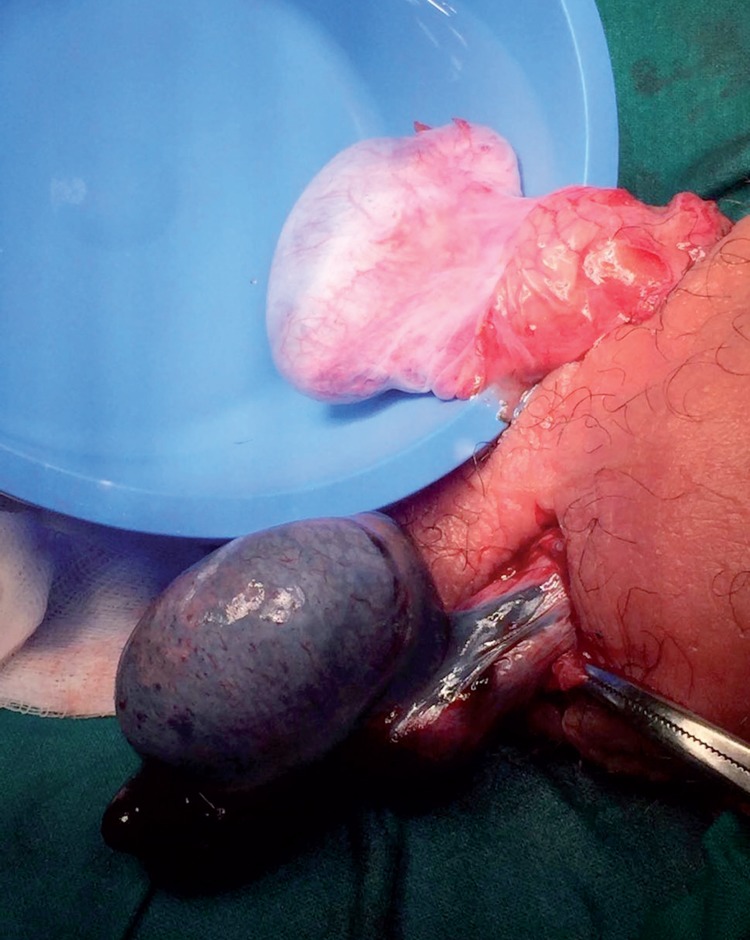
After bilateral detorsion, right testis recovered a normal coloration, in contrast of left testis with established necrosis.

The patient underwent immediate surgical exploration through a bilateral scrotal incision. First of all, we observed a 360° clockwise intravaginal torsion of the right testis, which presented a bluish coloration. Once detorsed, the testicle recovered a normal coloration and, accordingly, testis was fixed. Thereafter, we explored the left hemiscrotum observing a 720° torsed testis with necrotic appearance. Coloration slightly improved with detorsion but after waiting for 30 minutes, the testicle was considered nonviable, and a left orchiectomy was performed (Figure-2). A “bell clapper deformity” was observed on both testicles at the moment of surgical exploration. Pathological examination showed hemorrhagic necrosis of the testicle.

**Figure 3:**
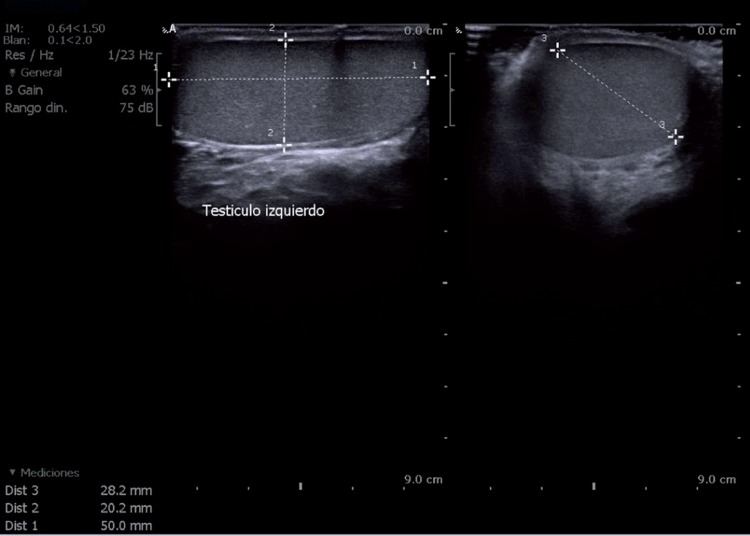
Ultrasound image one year after surgery. Right testis preserves a normal volume (14.8cc) and echogenicity.

One year after surgery, doppler ultrasound showed a right testicle with normal size (volume 14.8cc), echogenicity and vascularization (Figure-3). Serum sex hormone levels were within normal ranges.

## DISCUSSION

Testicular torsion is a surgical emergency with a common presentation in two age groups: adolescents and neonates (1). The vast majority of described cases for bilateral torsion were seen in neonates (2). Conversely, in adolescents and adults there are very few reported cases in the literature (four cases in adolescents (3-6) and five (7-11) in adults). Osada et al. (3) reported, in 1985, the first case of simultaneous bilateral testicular torsion in adolescent (12 years old), and Wasnick et al. (7) in 1985, the first case of bilateral torsion in an adult (24 years old).

The main peculiarity of our own case is that symptoms, physical examination and ultrasound imaging remarkably changed three hours after the initial evaluation. The two most important factors determining testicular damage are the time from the onset of symptoms and the degree of twisting of the cord. Tryfonas et al. (12) found absent or severely atrophied testis in all cases with torsion of more than 360° and symptoms duration beyond 24 hours. Similar results reported Wright (13), with either orchiectomy or atrophy in the follow-up in all patients with a 24-hour duration torsion. Based on this, when the patient came into the E.D. and was diagnosed of protracted unilateral torsion, we decided to perform an elective orchiectomy. However, the therapeutic approach suddenly changed with the diagnosis of bilateral testicular torsion, in order to prevent androgen deficiency and further infertility.

During surgical exploration, we observed that our patient presented a bilateral intravaginal torsion. There are two types of testicular torsion: intravaginal and extravaginal. Extravaginal torsion is mainly seen in neonates. During prenatal/neonatal period, it is common the presence of an undeveloped attachment of the tunica vaginalis to the scrotal wall, which explains the higher incidence of bilateral torsion in this age. As the child grows, the tunic attachments strengthen, reducing the likelihood of this type of torsion after the neonatal period (1). On the other hand, in older children and adults, the predominant type of torsion is intravaginal, in which is frequent to find anatomic abnormalities in the tunica vaginalis, mainly the “bell clapper anomaly”. In normal development, the tunica vaginalis surrounds the testicle except where the testicle attaches to the epididymis and the posterior scrotal wall. Thus, the posterior region of testicle is firmly attached to the scrotum. By contrast, in the “bell clapper anomaly”, the attachment of the tunica vaginalis to the testicle is inappropriately high, thus the spermatic cord can rotate within it and that can lead to an intravaginal torsion. This anatomical variant often is bilateral, which would explain our case and it permits to select those patients with an increased risk of bilateral torsion. Martin and Rushton (14) observed a negligible prevalence of contralateral “bell clapper deformity” in boys with previous unilateral neonatal torsion, however, in adolescents with testicular torsion, they found this deformity in the contralateral testicle in the majority of cases.

Finally, this report allows to emphasize that bilateral torsion presentation can be misleading and overlooked, which implies a careful physical examination of the contralateral testicle. Moreover, in cases of intravaginal torsion with associated “bell clapper deformity”, a contralateral testicular fixation could be justified.
